# Habenula bibliometrics: Thematic development and research fronts of a resurgent field

**DOI:** 10.3389/fnint.2022.949162

**Published:** 2022-08-03

**Authors:** Sifan Chen, Xiaoyu Sun, Yizhe Zhang, Yu Mu, Diansan Su

**Affiliations:** ^1^Department of Anesthesiology, Ren Ji Hospital, Shanghai Jiao Tong University School of Medicine, Shanghai, China; ^2^State Key Laboratory of Neuroscience, Center for Excellence in Brain Science and Intelligence Technology, Institute of Neuroscience, Chinese Academy of Sciences, Shanghai, China

**Keywords:** habenula, bibliometric, circuit, depression, p factor, therapeutic target

## Abstract

The habenula (Hb) is a small structure of the posterior diencephalon that is highly conserved across vertebrates but nonetheless has attracted relatively little research attention until the past two decades. The resurgent interest is motivated by neurobehavioral studies demonstrating critical functions in a broad spectrum of motivational and cognitive processes, including functions relevant to psychiatric diseases. The Hb is widely conceived as an “anti-reward” center that acts by regulating brain monoaminergic systems. However, there is still no general conceptual framework for habenula research, and no study has focused on uncovering potentially significant but overlooked topics that may advance our understanding of physiological functions or suggest potential clinical applications of Hb-targeted interventions. Using science mapping tools, we quantitatively and qualitatively analyzed the relevant publications retrieved from the Web of Science Core Collection (WoSCC) database from 2002 to 2021. Herein we present an overview of habenula-related publications, reveal primary research trends, and prioritize some key research fronts by complementary bibliometric analysis. High-priority research fronts include Ventral Pallidum, Nucleus Accumbens, Nicotine and MHb, GLT-1, Zebrafish, and GCaMP, Ketamine, Deep Brain Stimulation, and GPR139. The high intrinsic heterogeneity of the Hb, extensive connectivity with both hindbrain and forebrain structures, and emerging associations with all three dimensions of mental disorders (internalizing, externalizing, and psychosis) suggest that the Hb may be the neuronal substrate for a common psychopathology factor shared by all mental illnesses termed the p factor. A future challenge is to explore the therapeutic potential of habenular modulation at circuit, cellular, and molecular levels.

## Introduction

The habenula (Hb) is a phylogenetically conserved epithalamic nucleus connecting the forebrain with the ventral midbrain and hindbrain (Bianco and Wilson, 2009; Namboodiri et al., 2016). Evidence accrued over the past several decades indicates that the Hb contributes to cognitive behaviors in part as a negative regulator of the main monoaminergic systems (Nishikawa and Scatton, 1985; Nishikawa et al., 1986). Anatomically, the mammalian Hb is divided into the medial habenula (MHb) and lateral habenula (LHb), while the Hb of teleosts and amphibians is divided into ventral and dorsal regions (VHb and DHb) (Amo et al., 2010), each with distinct neuronal populations, input patterns, and projection targets (Aizawa et al., 2012). The Hb acts as a relay nucleus within which incoming cognitive information is processed and subsequently relayed to various other brain regions (Wang and Aghajanian, 1977; Matsumoto and Hikosaka, 2007; Bianco and Wilson, 2009). Based on these extensive connections, the Hb has been implicated in behaviors related to reward, sleep, aversive stimuli, anxiety, and fear (Matsumoto and Hikosaka, 2007; Geisler and Trimble, 2008; Fore et al., 2018). Consistent with a role as an anti-reward center, growing evidence now suggests that the hyperactivation of Hb, as a major component of networks, is one of the typical pathophysiological observations involved in the pathogenesis of depression, autism, schizophrenia, and drug abuse (Fakhoury, 2017; Cui et al., 2018; Yang et al., 2018; Buhler and Carl, 2021).

There has been a resurgence of interest in the Hb owing to advances in brain imaging, targeted ablation, and reversible stimulation and inhibition techniques, as well as new animal models (Proulx et al., 2014; Buhler and Carl, 2021; Lin et al., 2022). In the past decade, especially, a substantial number of high-quality publications on the habenula have appeared reporting new discoveries on physiological and potential pathophysiological functions (Hikosaka, 2010; Ootsuka and Mohammed, 2015; Ootsuka et al., 2017). As yet, however, there is no general conceptual framework of the Hb research field, and no studies have focused on uncovering potentially significant but overlooked topics. This article presents a scientometric overview of the habenula research field according to publications retrieved for the Web of Science Core Collection (WoSCC), and further reports qualitative and quantitative analyses of the current publication landscape, emerging trends, shifts in research fronts, and milestones using a variety of science mapping tools to identify current challenges and future directions.

## Materials and methods

### Data sources and search strategies

Relevant studies published from 2002 to 2021 inclusive were retrieved from the WoSCC database using the following query: TS = (habenu*), document type = Article AND language = English. We applied filters to limit the database index [Science Citation Index Expanded (SCI-EXPANDED)] and exclude article types (Proceedings Papers, Book Chapters, and Data Papers). Study retrieval and data downloads were completed in 1 day (February 20, 2022) to reduce potential internal inconsistencies due to database updates.

### Data collection

Publication metadata were extracted and recorded for title, publication year, author details, country, institution, journal, citation number, and full citation profile. After reading the title and abstract of each manuscript, those focusing on the habenula were downloaded in .txt format and subsequently imported into a science mapping tool for further analysis.

### Data analysis and visualization

Bibliometric analyses were conducted primarily using CiteSpace V5.8.R3, 64 bit (Drexel University, Philadelphia, PA, United States) (Chen, 2006), VOSviewer 1.6.15 (Leiden University, Leiden, Netherlands) (Perianes-Rodriguez et al., 2016), Bibliometrix (Aria and Cuccurullo, 2017), and the Bibliometry online analysis platform^1^ (Chen et al., 2020). We applied several bibliometric indicators such as impact factor (IF) and category quartile (from Journal Citation Reports 2020) for quantification of publication repercussion, the PlumX Metrics for academic impact on social media, and Bradford’s law to map the journal distribution. Annual publication output, growth tendencies, and citation profiles of countries, institutions, and individual authors were analyzed using the Bibliometry online platform and the R package Bibliometrix. Prime bibliometric analyses of document co-citations, collaboration networks, and keyword bursts were conducted using CiteSpace to identify knowledge domain and emergent research fronts. A co-occurrence network of keywords was generated using VOSviewer. The detailed parameter settings for each software application are provided in Supplementary material.

To identify additional emergent research fronts and validate others, we searched for relevant current and past clinical studies registered at ClinicalTrials.gov. Finally, we downloaded the latest publications on Hb in the past 5 years (2018–2022) and analyzed the top cited articles and highest-ranking references in the co-citation network to help identify emerging concepts.

## Results

### Overview of habenula-related publications

**FIGURE 1:**
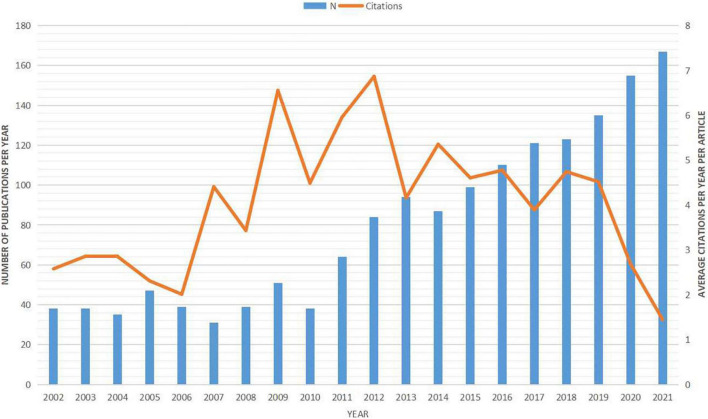
Number of annual publications on the habenula from 2002 to 2021. Bars represent the annual publication number (left) and line represents the average citation number per year per documents (right).

After removing mismatched data, a total of 1,608 articles were retained for analysis. In total, these studies were cited 50,980 times, for an average of 31.70 citations per article. The number of yearly publications increased at an average annual growth rate of 8% from 2002 to 2021. Over this same period, the annual citation number per publication fluctuated from 1.45 to 6.55 (Figure 1). Knowledge mapping was then conducted to identify the most influential scholars and research groups as well as the network of collaborators. Supplementary Table 1 summarizes the top 10 most active countries/regions contributing to Hb research. The largest number was conducted in the United States (*n* = 721, 44.62%), followed by China (*n* = 214, 13.24%). Cooperative analysis of countries/regions was shown in Supplementary Figure 1A. Of the 10 most productive research affiliations, five are located in the United States, three in China, and one each in Germany and France (Supplementary Table 1). The collaboration network of affiliations indicates that research groups are relatively sparsely distributed (density = 0.0065) (Supplementary Figure 1B), indicating few local collaborations or geographic concentrations of collaborative groups. Over the past 20 years, studies related to the Hb have been published in 331 journals, the top 11 of which are listed in Supplementary Table 2. The highest-ranked journal according to total number of citations was Journal of Neuroscience with 1,662, followed by the Journal of Comparative Neurology with 896. Construction of a Bradford journal distribution map (Supplementary Figure 1C) confirmed these top 11 as “core” journals, as all were within the central region of the map including 34.39% (553/1608) of publications. In total, 6,632 authors contributed to the 1,616 publications identified. The top 25 most productive authors are listed in Supplementary Table 3. The collaborative network of authors generated using CiteSpace includes 657 nodes and 944 links (Supplementary Figure 1D).

### Keyword co-occurrence analysis

**FIGURE 2:**
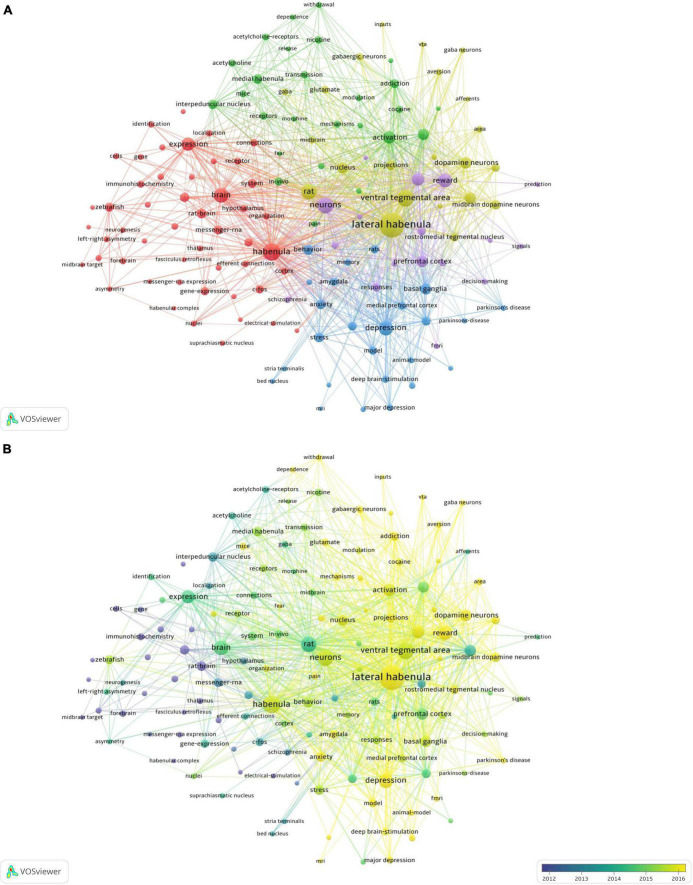
Co-occurrence network analysis of keywords (*n* = 140) appearing at least 20 times. **(A)** Classification into five clusters. **(B)** Average publication year for each cluster.

A total of 140 author keywords and Keywords Plus with at least 20 occurrences were identified by VOSviewer (Figure 2). In this figure, the size of the bubble represents the number of occurrences and the different colors represent the cluster and average publication year. The most studied topics related to Hb included lateral habenula (577), ventral tegmental area (256), depression (182), reward (178), dopamine (144), activation (139), substantia nigra (118), prefrontal cortex (116), basal ganglia (111), and nucleus accumbens (108), while the most recent emergent topics were circuits (average publication year of 2019), fear (2018), dependence (2018), aversion (2018), major depressive disorder (2018), and anxiety (2017).

### Top 10 cited articles and plum X metrics analysis

The ten articles with the highest global citation numbers are ranked in Table 1. An article by Masayuki and Hikosaka in Nature (2007) ranked first with 843 citations, followed by a Nature article by Lammel and colleagues (812 citations). Notably, Masayuki co-authored three articles published in 2007 and 2009 that received 843, 807, and 343 citations. These top cited articles are often regarded as landmarks in Hb research. PlumX is one of the most popular altmetric indicators, reflecting timely, and multidimensional impacts of an academic publication, especially its influences *via* social media (Lindsay, 2016). Plum X metrics of the highest cited articles were extracted on March 15, 2022. The article by Masayuki and Hikosaka in Nature (2007) was the most influential document, ranking first in three out of five categories, while studies by Lammel and colleagues and Bo and colleagues scored highest in Captures and Usage categories, respectively.

**TABLE 1:** The top 10 high-cited papers in habenula research during 2002–2021.

Rank	Title	Journal	Author	Publication year	Total citations	PlumX metrics				
						Citations	Usage	Captures	Mentions	Social media
1	Two types of dopamine neuron distinctly convey positive and negative motivational signals	Nature	Matsumoto, Masayuki	2009	843	869	1,355	1,541	3	76
2	Input-specific control of reward and aversion in the ventral tegmental area	Nature	Lammel, Stephan	2012	812	844	1,182	1,596	1	25
3	Lateral habenula as a source of negative reward signals in dopamine neurons	Nature	Matsumoto, Masayuki	2007	807	824	854	1,097	2	/
4	Habenular alpha 5 nicotinic receptor subunit signalling controls nicotine intake	Nature	Fowler, Christie D	2011	427	457	2,642	357	2	1
5	The rostromedial tegmental nucleus (RMTg), a GABAergic afferent to midbrain dopamine neurons, encodes aversive stimuli and inhibits motor responses	Neuron	Jhou, Thomas C	2009	424	434	249	532	/	/
6	Error monitoring using external feedback: Specific roles of the habenular complex, the reward system, and the cingulate motor area revealed by functional magnetic resonance imaging	Journal of Neuroscience	Ullsperger, M	2003	377	393	1	438	1	1
7	A prefrontal cortex-brainstem neuronal projection that controls response to behavioural challenge	Nature	Warden, Melissa R	2012	374	392	117	994	1	19
8	Synaptic potentiation onto habenula neurons in the learned helplessness model of depression	Nature	Li, Bo	2011	385	389	4,507	721	/	20
9	GABA neurons of the VTA drive conditioned place aversion	Neuron	Tan, Kelly R	2012	364	377	63	731	1	5
10	Representation of negative motivational value in the primate lateral habenula	Nature Neuroscience	Matsumoto, Masayuki	2009	343	344	853	524	1	/

### Document co-citation and cluster analysis

**FIGURE 3:**
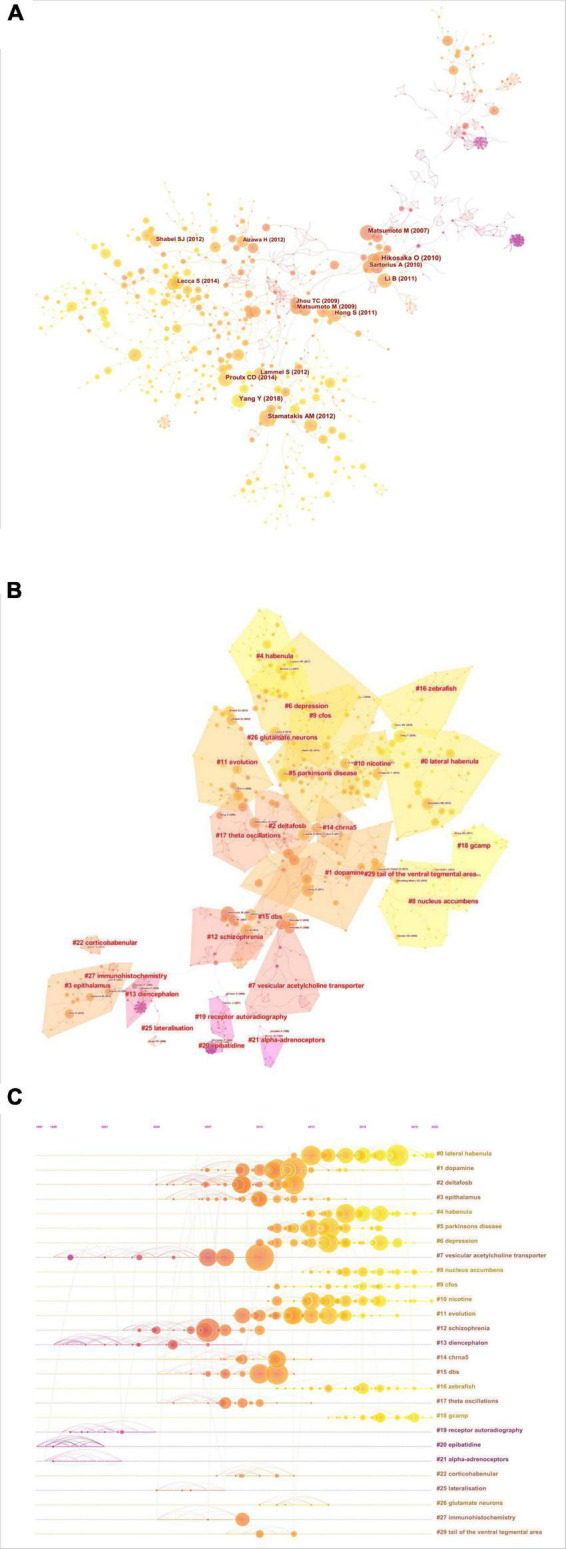
Co-citation network of references. **(A)** Co-citation map of references on the habenula. **(B)** Clustered network map of co-cited references on the habenula. **(C)** Timeline view of co-citation clusters with cluster labels shown on the right.

A co-citation network based on the 54,479 references from the 1608 included documents was then constructed using CiteSpace in a hierarchical order. This final network consists of 1,015 nodes and 1,612 links (Figure 3A), and is displayed with node size proportional to co-citation frequency and time of citation by tree-ring colors. As shown in Figure 3B, the co-cited references were grouped into twenty-seven major clusters (lateral habenula, dopamine, DeltaFos-B, epithalamus, habenula, Parkinson’s disease, depression, vesicular acetylcholine transporter, nucleus accumbens, c-Fos, nicotine, evolution, schizophrenia, diencephalon, CHRNA5, DBS, zebrafish, θ oscillations, GCaMP, receptor autoradiography, epibatidine, a-adrenoceptors, corticohabenular, lateralization, glutamate neurons, immunohistochemistry, and tail of the ventral tegmental area). The modularity score was 0.9125 and the silhouette score 0.9707, indicating remarkably high cluster clarity and homogeneity. Cluster labels were generated based on the word profiles derived from the citing articles of the reference cluster. Figure 3C displays a timeline view of each co-citation cluster. Cluster #0 (lateral habenula), Cluster #6 (depression), Cluster #8 (nucleus accumbens), Cluster #9 (c-Fos), Cluster #10 (nicotine), Cluster #16 (zebrafish), and Cluster #18 (GCaMP) had the most recent citation bursts and so are indicative of current research fronts.

### Burst detection

**FIGURE 4:**
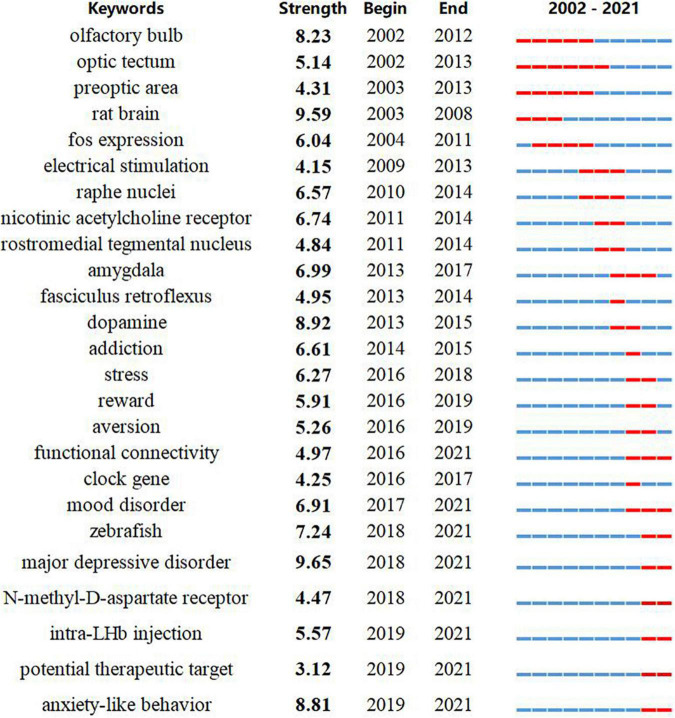
Keywords with the strongest citation bursts in original articles between 2001 and 2021. The timeline is depicted as a year-sliced blue line, and the time interval of a burst is marked as a red section on the blue timeline to indicate the beginning/ending year and the duration of a citation burst.

Burst detection was applied to identify rapid increases of interest in specific habenula research topics using the bursting keywords algorithm in CiteSpace (Chen, 2006). Keyword burst detection revealed several emergent topics. After manually excluding keywords with little specific association with the Hb, we identified the 25 with strongest bursts and thus representative of the research trends (Figure 4). Throughout the entire study period from 2002 to 2021, major depressive disorder exhibited the highest burst strength (9.65), and was also the most recent bursty term. Notably, there was a distinct transition in research themes from circuit connections (olfactory bulb, optic tectum, preoptic area, rostromedial tegmental nucleus, etc.) to physiological functions (stress, reward, and aversion) and ultimately to pathophysiological conditions (mood disorder, major depressive disorder, and anxiety-like behavior). Moreover, there was also a trend for a switch in animal models from rodents (“rat brain,” 2003–2008) to non-mammalian vertebrates (“zebrafish,” 2018–2021). Intriguingly, the last three terms with strong bursts, *N*-methyl-*D*-aspartate (NMDA) receptor, intra-LHb injection, and potential therapeutic target are related to potential therapeutic roles of the habenula, and this association was also found for search results from the ClinicalTrials.gov.

### Analysis of the most recent documents and ClinicalTrials.gov

**FIGURE 5:**
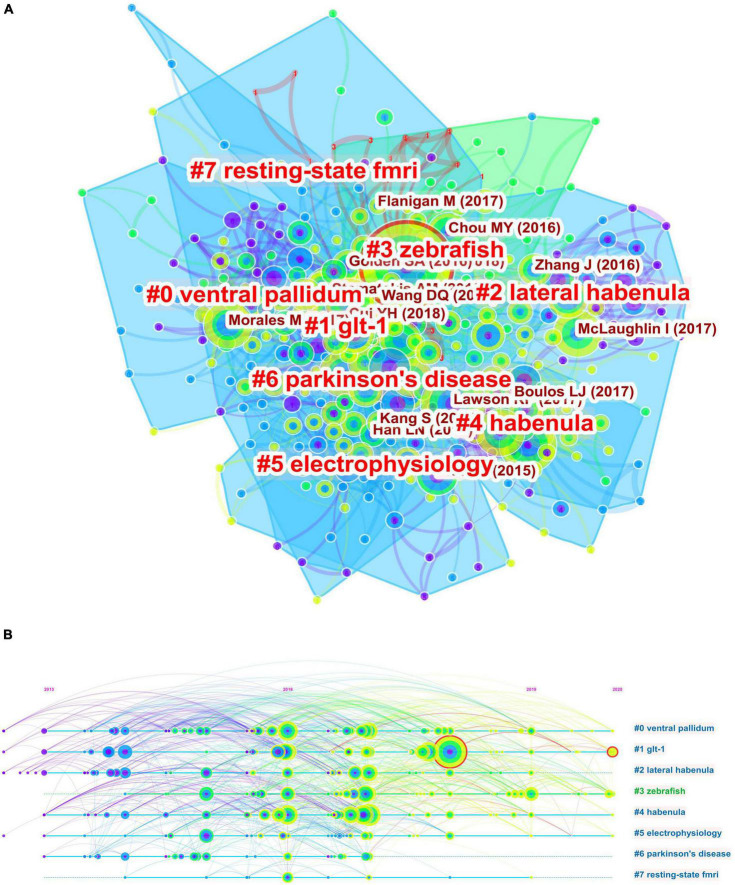
Co-citation analysis of publications from the past 5 years. **(A)** Co-citation clustering of references on the habenula. **(B)** The timeline view of co-citation clusters with cluster labels shown on the right.

In order to fully characterize the major current research fronts, we conducted separate bibliometric analyses for the most recent 5 years (2017–2022). A total of 618 articles were obtained using the same search query, and co-citation analysis was used to identify cluster labels and bursts for this recent body of references. Figures 5A,B show the co-citation clustering and timeline view. Cluster #1 related to GLT-1 had the strongest citation burst, and ventral pallidum, GLT-1, and Parkinson’s disease were judged as recent research fronts. Table 2 lists the most influential references in the past 5 years as ranked by sigma and centrality values, which are measures of scientific novelty and structural significance, respectively (Chen et al., 2010). A study by Proulx et al. (2014) in Nature Neuroscience had the highest sigma value at 1.34, while another by Yang and colleagues (Yang et al., 2018) in Nature ranked the first in terms of centrality value (0.14).

**TABLE 2:** High-impact references in the co-citation network of habenula research between 2017 and 2022 (ranked by sigma and centrality).

Rank	Title	Journal	Author	Publication year	Sigma
1	Reward processing by the lateral habenula in normal and depressive behaviors	Nature Neuroscience	Christophe D Proulx	2014	1.34
2	Neuronal dynamics regulating brain and behavioral state transitions	Cell	Aaron S Andalman	2019	1.18
3	Circuit architecture of VTA dopamine neurons revealed by systematic input-output mapping	Cell	Kevin T Beier	2015	1.15
4	Comprehensive identification and spatial mapping of habenular neuronal types using single-cell RNA-seq	Current Biology	Shristi Pandey	2018	1.13

**Rank**	**Title**	**Journal**	**Author**	**Publication year**	**Centrality**

1	Ketamine blocks bursting in the lateral habenula to rapidly relieve depression	Nature	Yan Yang	2018	0.14
2	Shifted pallidal co-release of GABA and glutamate in habenula drives cocaine withdrawal and relapse	Nature Neuroscience	Frank J Meye	2016	0.12
3	Transcriptomic-anatomic analysis of the mouse habenula uncovers a high molecular heterogeneity among neurons in the lateral complex, while gene expression in the medial complex largely obeys subnuclear boundaries	Brain Structure and Function	Brain Structure and Function	2016	0.12

In addition to co-citation clustering and burst detection, we conducted another search on March 14, 2022 and found six clinical trial reports related to the habenula that have been antecedently registered at ClinicalTrials.gov (Table 3). Five of these clinical trials investigated deep brain stimulation (DBS) of the habenula, four for treatment-resistant major depressive disorder (NCT03347487, NCT01798407, NCT03254017, and NCT03667872) and one for obsessive-compulsive disorder (NCT03463590). Another trial was set to investigate the effects of ketamine on learning and habenular neural activity changes as measured by blood oxygen level dependent (BOLD) signals in healthy volunteers. Collectively, these analyses indicate a progressive shift from anatomic, physiological, and pathophysiological studies to clinical applications targeting the habenula.

**TABLE 3:** Clinical trials exploring habenula that were registered with ClinicalTrials.gov.

NCT number	Research theme	Status	Study title	Conditions	Sample size (n)	Study type	Study design
NCT03347487	DBS	Terminated	DBS of the habenula for treatment-resistant major depression	Treatment resistant major depression disorder	7	Interventional	Allocation: N/A| Intervention Model: Single Group Assignment| Masking: None (Open Label)| Primary Purpose: Treatment
NCT03463590	DBS	Unknown status	Deep brain stimulation of the bilateral habenula for treatment-refractory obsessive-compulsive disorder	Obsessive-compulsive disorder	6	Interventional	Allocation: N/A| Intervention Model: Single Group Assignment| Masking: None (Open Label)| Primary Purpose: Treatment
NCT01798407	DBS	Active, not recruiting	DBS of the lateral habenula in treatment-resistant depression	Treatment resistant major depressive disorder	6	Interventional	Allocation: Non-Randomized| Intervention Model: Sequential Assignment| Masking: Quadruple (Participant, Care Provider, Investigator, Outcomes Assessor)| Primary Purpose: Treatment
NCT03254017	DBS	Terminated	Remotely programmed deep brain stimulation of the bilateral habenula for treatment- resistant major depression: an open label pilot trial	Treatment resistant major depressive disorder	2	Interventional	Allocation: N/A| Intervention Model: Single Group Assignment| Masking: None (Open Label)| Primary Purpose: Treatment
NCT03667872	DBS	Not yet recruiting	Efficacy and safety of DBS in patients with treatment-resistant depression	Treatment resistant depressive disorder	6	Interventional	Allocation: N/A| Intervention Model: Single Group Assignment| Masking: None (Open Label)| Primary Purpose: Treatment
NCT04850911	Ketamine	Not yet recruiting	Reward emotion learning and Ketamine study	Depression| Major depressive Disorder| Treatment resistant depression	35	Interventional	Allocation: Randomized| Intervention Model: Parallel Assignment| Masking: Double (Participant, Investigator)| Primary Purpose: Basic Science

## Discussion

### Trends in habenula research

Bibliometric mapping was implemented to summarize progress in habenula research from 2002 to 2021. Based on integrative visualization and analysis using four complementary science mapping tools, we qualitatively and quantitatively depict the intellectual landscape, top research topics, and the emerging research fronts in habenula research. Since the 1970s and 1980s, there has been growing interest in the habenula, but this has been hampered by the perception that it has no well-defined function roles (Herkenham and Nauta, 1977; Wang and Aghajanian, 1977; Cuello et al., 1978; Christoph et al., 1986). Due to seminal work showing that the LHb is a key regulator of negatively motivated behavior (Matsumoto and Hikosaka, 2007, 2009a,b; Jhou et al., 2009) and more recent evidence of efficacy as a therapeutic target for major psychiatric disorders (Yang et al., 2008; Li et al., 2011, 2013), the number of scientific publications is now growing steadily at about 8% per year. To date, multidisciplinary findings demonstrate extensive connectivity of the habenula with the rest of the brain and intrinsic heterogeneity contributing to diverse physiological and pathophysiological processes (Benarroch, 2015; Boulos et al., 2017). However, no study has provided a comprehensive bibliographic analysis of landmark publications, the progress of different subfields, and emerging research topics. Our goal was to catalog the attributes of related studies and thereafter discussed about the results of cluster labeling and burst detection to predict and advance the future studies.

While the overall scientific output has increased from 2002 to 2021, the first 9 years were relatively stable in terms of publication numbers, underscoring recent growth in interest. Using burst detection, we specifically identified references with sharp increases in citation frequency within specific time periods. This analysis indicated that several articles prior to 2010 have had major influences on the subsequent growth and trajectory of Hb research. Matsumoto and Hikosaka (2007) reported that the LHb is a major source of negative reward signals to dopamine neurons, while Ji and Shepard (2007) concluded that LHb stimulation induces transient suppression of midbrain dopamine neuron activity *via* a GABA_A_ receptor-dependent mechanism. In the same year, Lecourtier and Kelly (2007) summarized the critical role of the habenular complex in regulating monoaminergic systems and cognitive behaviors. We detected three peak years for average citation number, 2007, 2009, and 2012, with 6 of the top 10 cited articles appearing in 2009 and 2012 (Table 1; Jhou et al., 2009; Matsumoto and Hikosaka, 2009a,b; Lammel et al., 2012; Tan et al., 2012; Warden et al., 2012). Likewise, reference burst detection identified several studies published in 2009 and 2012 with burstiness values > 20 (Kaufling et al., 2009; Shabel et al., 2012; Stamatakis and Stuber, 2012; Supplementary Figure 2). These studies are now considered seminal in habenula research.

### Research fronts of the habenula

#### Overview

In bibliometric terms, research front of a given field refers to a group of citing articles while the intellectual base was composed of the co-cited references (Chen et al., 2010). Analyzing the word profiles extracted from articles citing a cluster of references yields co-citation cluster labels that were then used to clarify and interpret the major concepts of the research front. Key concepts derived from co-citation analysis included *lateral habenula, depression, nucleus accumbens, nicotine, zebrafish*, and *GCaMP*, while separate co-citation analysis the most recent references included *ventral pallidum, GLT-1, and Parkinson’s disease*. By combining these concepts with burst detection, a method for detecting emergent terms independent of host article citation times, we were able to identify and clarify research fronts or emerging concepts at both structural and temporal levels. Burst detection of keywords based on frequency of occurrence yielded keywords such as *zebrafish, major depressive disorder, NMDA receptor, potential therapeutic target*, and *anxiety-like behavior*. Surprisingly, we found some research themes from keywords burst detection were corresponding to the searching results from the ClinicalTrials.gov. Also, burst detection of citations from the most recent publications (2017–2022) identified documents that have attracted the most attention of peer scholars. Here we briefly discuss how emergent research fronts have advanced the field in promising new directions and address potentially fruitful future directions. As a recent comprehensive review summarized potential functions of the lateral habenula in various brain networks and major psychiatric disorders (Hu et al., 2020), while others have discussed the importance of the medial habenula in these areas (Viswanath et al., 2014; Lee et al., 2019), we focus on more recent findings and related topics identified as research fronts based on our bibliometric analysis.

#### Circuitry connection

**FIGURE 6:**
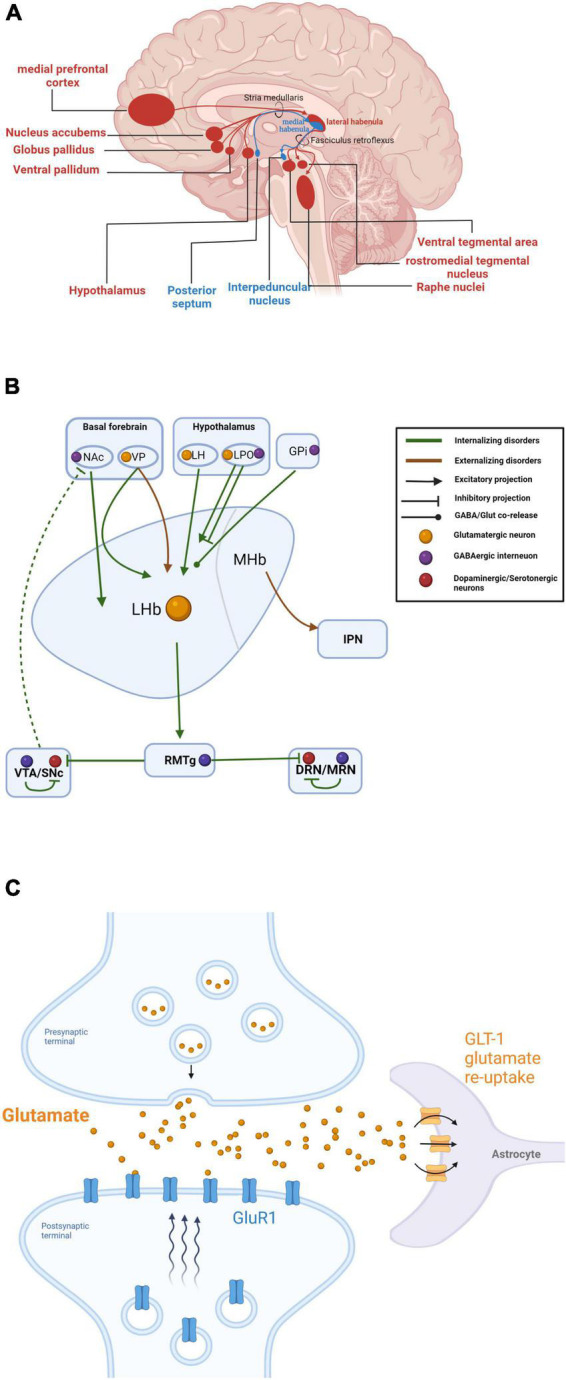
**(A)** Summary of habenular anatomical circuitry. Inputs and outputs to and from the lateral and medial habenula are shown in red and blue, respectively. **(B)** Summary of habenular circuits related to different dimensions of psychiatric disorders. Internalizing disorders (such as major depressive disorders and anxiety disorders) are preferentially related to upstream projections to the Hb from the nucleus accumbens (NAc), ventral pallidum (VP), lateral hypothalamus (LH), and lateral preoptic area (LPO), and downstream projections to the rostromedial tegmental nucleus (RMTg), ventral tegmental area (VTA)/substantia nigra pars compacta (SNc), and dorsal raphe nucleus (DRN)/medial raphe nucleus (MRN). Externalizing disorders are exclusively related to the ventral pallidum (VP) and interpeduncular nucleus (IPN). **(C)** Mechanism for GLT-1 regulation of extracellular glial glutamatergic transmission and LHb neuron bursting. Created with BioRender.com.

Numerous studies have been conducted to reveal the circuitry of the habenula. The MHb receives afferent glutamatergic inputs primarily from the posterior septum (PS), while its major downstream target is the interpeduncular nucleus (IPN) (Viswanath et al., 2014; Otsu et al., 2018). In contrast, the LHb has more distributed connections, acting as a hub linking various structures in the limbic areas, basal ganglia, and medial prefrontal cortex (mPFC) to the GABAergic rostromedial tegmental nucleus (RMTg) (Herkenham and Nauta, 1977; Hu et al., 2020; Figure 6A). While many of this connection were described decades ago, the knowledge of the diverse connections to the habenula in regard to its well-established function was relatively limited.

##### Ventral pallidum

The ventral pallidum (VP) is a cytologically heterogeneous region of the basal ganglia implicated in behavioral responses to both rewarding and aversive stimuli. Though VP neurons are predominantly GABAergic, studies by Faget et al. (2018) and others (Stamatakis and Stuber, 2012; Knowland et al., 2017; Tooley et al., 2018) have suggested that VP glutamatergic neurons project in greater abundance to the LHb, resulting in increased LHb neuronal activity upon VP activation. Notably, optogenetic activation of this glutamatergic VP-LHb circuit was found to induce depressive-like behaviors in rats (Liu et al., 2020). Subsequent studies have also suggested function roles of the VP-Hb pathway in salience processing (Wang F. et al., 2020), generating feelings of disgust (Khan et al., 2020) and cocaine seeking (Pribiag et al., 2021).

##### Nucleus accumbens

The nucleus accumbens (NAc) is the classic pleasure and reward locus of the basal forebrain (Salgado and Kaplitt, 2015). Golden et al. (2016) suggested that descending GABAergic projections from the NAc to LHb encode reward associated with aggression. Reciprocally, the LHb was found to modulate the NAc through indirect inhibition of dopamine (DA) release into the NAc from the ventral tegmental area (VTA) (Lecourtier et al., 2008; Ostroumov and Dani, 2018). It is noteworthy that the rostromedial tegmental nucleus (RMTg), a GABAergic nucleus downstream of the LHb, sends the densest (and most studied) inhibitory input to VTA DA neurons and is regarded as a major source of relayed aversive signals from the LHb (Brown et al., 2017). Given that the NAc is a crucial site mediating reward-related behavior, it is rational to expect that the LHb regulates positive and aversive behavioral consequences through the LHb-NAc circuit. However, links between these regions has remained elusive. A recent study has implicated a direct link between perihabenular nucleus (pHb) and NAc in mediating light-at-night (LAN) induced depression (An et al., 2020). In contrast to previous investigation that mPFC was the most innervated downstream site of pHb (Fernandez et al., 2018), NAc was prominent in conveying retinal-specific signals, which expanded the role of NAc as a downstream site of Hb.

#### Physiological properties of the habenula

##### Nicotine and medial habenula

The Hb is a critical relay hub providing top-down control of forebrain and midbrain aminergic nuclei, and it is widely believed that this nodal position allows the Hb to regulate neurological processes associated with aminergic signaling, including behavioral responses to rewarding and aversive stimuli (Hikosaka, 2010; Proulx et al., 2014), motivation (Hikosaka et al., 2008), cognition (Mathis et al., 2017; Nuno-Perez et al., 2021), circadian rhythms (Bano-Otalora and Piggins, 2017; Young et al., 2022), and pain (Shelton et al., 2012). Compared to the LHb, the MHb is little studied, but recent findings suggest that it is a major source of cholinergic circuits in the brain (Lopez et al., 2019). According to our co-citation analysis, the medial habenula cholinergic system has attracted current attention because of its potential relationship between the MHb and nicotine withdrawal, drug addiction, and mood disorders (McLaughlin et al., 2017; Lee et al., 2019). Moreover, MHb-IPN (interpeduncular nucleus) pathway showed left-right functional asymmetry in the level of neurotransmitter acetylcholine (Hong et al., 2013), which also revealed the involvement of this pathway in nicotine addiction (Souter et al., 2022). Notably, the CHRNA5 gene encoding the nicotinic receptor alpha 5 subunit, which is strongly implicated in smoking dependence, is expressed at highest mRNA levels in the IPN, while the A3/B5 genes encoding channel-forming α3 and β4 receptor subunits are prominently expressed in the ventral MHb (Morton et al., 2018). Recent studies have expanded our views on how the habenula regulates nicotine intake and aversion. The orphan G-protein-coupled receptor (GPCR)151 is enriched in both rodent and human MHb and can influence nicotine responses by reducing cyclic adenosine monophosphate (cAMP) levels and thereby suppressing synaptic neurotransmission within the MHb-IPN pathway (Antolin-Fontes et al., 2020). This emerging picture may guide future studies exploring the potential of the MHb-IPN pathway as a therapeutic target for drug abuse.

##### GLT-1

The LHb is hyperactivated during depression, and recent findings suggest a potential contribution of local glial cells (Cui et al., 2018). Functional changes in the glial glutamate transporter GLT-1, which tightly regulates extracellular glial glutamate transmission, were found to modulate the firing rate of LHb neurons and elicited depressive-like phenotypes in rodents (Cui et al., 2014; Kang et al., 2018; Aizawa et al., 2020; Figure 6C), and a more recent study found downregulation of GLT-1 in the LHb after lesioning the substantia nigra pars compacta (SNc) in a rat model of Parkinson’s disease (PD) (Lyu et al., 2021). Although current evidence supported that the glial GLT-1 in the LHb was closely related to depression, how GLT-1 malfunction affects depressive-like behaviors in animal models remains to be shown.

##### Zebrafish and GCaMP

The zebrafish larva has emerged as a valuable model for studies of Hb function owing to its highly conserved brain structure, well-described neuromodulatory systems, translucency for neuroimaging study, and genetic accessibility (Mu et al., 2020). Particularly valuable recent developments include genetically encoded calcium indicators (GECIs) such as GCaMP that allow for non-invasive whole-brain imaging at high spatiotemporal resolution (Nakai et al., 2001; Muto and Kawakami, 2011; Randlett et al., 2015), single-cell RNAseq analysis in the zebrafish Hb that provides comprehensive molecular foundation of habenula physiology (Pandey et al., 2018) and the availability of genetic resources and gene-manipulation tools such as GAL4:UAS (Scott et al., 2007), Tol2 (Asakawa et al., 2008), and CRISPR-Cas system (Hwang et al., 2013) could be possible reasons for the recent shifting from mammalian models to zebrafish. Notably, zebrafish Hb has comparatively large size and high accessibility, which also facilitates more precise circuit dissection studies of Hb. Using *in vivo* Ca^2+^ imaging, several studies have demonstrated that the sensory responses of dHb neurons are asymmetric and subpopulation-specific. The left and right dHb neurons preferentially responded to visual and olfactory cues, respectively (Jetti et al., 2014; Zhang et al., 2017; Chen et al., 2019; Choi et al., 2021). New imaging approaches (Ahrens et al., 2013; Voleti et al., 2019; Zhang Z. et al., 2021) as well as novel biosensors (Patriarchi et al., 2018; Mohr et al., 2020; Sha et al., 2020) may facilitate studies on the larger scale neural basis of habenula-related behaviors.

#### The habenula as a potential therapeutic target

The extensive connectivity and intrinsic heterogeneity of the habenula likely contribute to the multiple pathophysiological changes and diverse behavioral phenotypes (Benarroch, 2015; Boulos et al., 2017). Notably, the importance of Hb for various psychiatric symptoms and disorders has been actively debated for years. However, this anatomic and functional diversity has made it difficult to construct a general framework for fully understanding Hb contributions to psychiatric disorders. Here we also noticed recent controversies in psychiatric nosology, questioning the continuity rather than discreteness of symptoms between disorders, as for the psychiatric comorbidity rates were surprisingly high (Kotov et al., 2017). Caspi and Moffitt proposed a more generalized innate factor, denoted as general factor of psychopathology or p factor, to summarize and indicate positive correlation among all psychiatric comorbidities (Caspi and Moffitt, 2018). Current evidence strongly suggests that that the Hb links all three dimensions of these disorders, including major depressive disorders, and anxiety disorders (internalizing) (Cui et al., 2018; Kang et al., 2018), substance use disorders (externalizing) (McLaughlin et al., 2017), and bipolar disorders and schizophrenia (psychotic experiences) (Figure 6B; Zhang C. et al., 2019; Wang Y. et al., 2020). Therefore, habenula may offer the greatest promise of being the neuronal substrate of p factor (also reviewed in Lee and Goto, 2021). Thus, interventions targeting the Hb may show efficacy against multiple disorders. Here, we briefly summarize studies on interventions targeting the habenular for treatment of multiple psychiatric disorders.

##### Ketamine as a rapid-acting antidepressant

Ketamine, although first introduced as an anesthetic drug, burst on its scene as a rapid anti-depressant drug since 2000 (Berman et al., 2000) and soon emerged as a new approach beyond the traditional concepts of momoamine-based antidepressant therapy (Sial et al., 2020). Ketamine uncompetitively inhibits NMDA-type ionotropic glutamate receptors, thereby preventing cation influx, postsynaptic depolarization, and synaptic plasticity. These neural responses are associated with rapid and profound antidepressant effects in model animals and human patients (Paoletti and Neyton, 2007; Hashimoto, 2019). Further, the habenula has been reported to be the only brain region showing persistent activation in different animal models of depression (Caldecott-Hazard et al., 1988; Andalman et al., 2019). The idea that the habenula mediates the antidepressant effects of ketamine is not new, but its mechanism was identified only recently by Yang et al. (2018). The habenula-centric hypothesis of ketamine antidepressant properties, while intriguing, is complicated since ketamine was considered as a promiscuous drug (Zanos et al., 2018). Nonetheless, unraveling the circuits underlying these antidepressant effects may lead to the development of novel rapid-acting antidepressant drugs without behavioral side effects.

##### Deep brain stimulation for treatment-resistant depression

Deep brain stimulation (DBS) is a new therapeutic approach for treatment-resistant depression (TRD) (Holtzheimer et al., 2017; Dandekar et al., 2018; Drobisz and Damborska, 2019) and other psychiatric disorders (Harmsen et al., 2020). In the past decade, a series of preliminary clinical studies has explored the efficacy and safety of Hb-DBS for treatment-refractory psychiatric disorders (Yadid et al., 2013; Zhang C. et al., 2019, 2022; Wang Y. et al., 2020; Zhang C. et al., 2021). Despite the limited sample size in each of the study and the primary disadvantages of DBS such as poor spatial resolution and invasiveness, therapeutic benefits of Hb-DBS were generally consistent throughout the various studies and pointed out the encouraging and promising potential of Hb-DBS as a viable therapeutic target. Future studies with larger enrollment, better sham-control design, variability-adaptation, and precise randomization will be essential to firmly establish the clinical implications and benefits of Hb-DBS.

##### Potential drug targets in habenula

G-protein-coupled receptors (GPCRs) are common targets of current pharmaceutical drugs (McCusker et al., 2007; Santos et al., 2017). One such GPCR, GPR139, was found to be enriched in the medial habenula and highly conserved across species (Susens et al., 2006; Hu et al., 2009; Liu et al., 2015). Studies using GRP139 agonists (Kononoff et al., 2018; Reichard et al., 2021) or antagonists (Hu et al., 2009) suggest that GPR139 signaling contributes to substance dependence in animal models. Intriguingly, GPR139-positive MHb neurons send projections to the IPN, a pathway suggested regulating nicotine withdrawal and drug abuse. Moreover, a recent study reported potential functions in Parkinson’s disease (Andersen et al., 2016), schizophrenia (Dao et al., 2022), and analgesia (Wang et al., 2019). Notably, a phase I clinical study of the potent and selective GPR139 agonist TAK-041 demonstrated good tolerance in both healthy volunteers and patients with stable schizophrenia (Yin et al., 2022).

## Conclusion

To our knowledge, this is the first study to provide a general conceptual framework for the habenula research field. Using multiple bibliometric tools, we quantitatively and qualitatively analyzed the existing publication landscape, visualized the research trends, and prioritized some key research fronts. Over the past 20 years, the research themes of habenula have undergone radical changes. Detailed investigation on the physiological roles of the habenula in the first few years of this period suggested relevance to multiple psychiatric disorders, and current research fronts are exploring the therapeutic potential of habenula at circuit, cellular, and molecular levels.

## Data availability statement

The original contributions presented in this study are included in the article/Supplementary material, further inquiries can be directed to the corresponding author.

## Author contributions

SC and DS: study design. SC, XS, and YZ: data acquisition and analysis. SC: manuscript drafting and tables and figures making. SC, YM, YZ, and XS: manuscript revising and modifying. All authors read and approved the final manuscript.

## Footnotes

1
http://bibliometric.com/
