# Supplementary material for: Habenula bibliometrics: Thematic development and research fronts of a resurgent field
Source: Front Integr Neurosci. 2022 Aug 3;16:949162. doi: 10.3389/fnint.2022.949162 (PMC9382245; doi:10.3389/fnint.2022.949162)
Supplement: Supplementary file 1 [file Data_Sheet_1.DOCX]

Full records and cited references of related publications were downloaded from the WoSCC database, saved in.TXT format, and then imported into the CiteSpace software V5.8.R3, 64bit (Drexel University, Philadelphia, PA, USA), and the following options were used: the time-slicing was set to “2001–2020”; the number of years per slice was set to “1”; the selection criterion was set to “g-index”; and the scale factor k was set to “25”; moreover, the options “pathfinder” and “pruning the merged network” were selected in order to reduce the number of links while retaining the most salient structure; for the node type, only one option was selected at a time from “author,” “institution,” “country,” “reference,” “cited author,” and “keyword.” VOSviewer 1.6.15 (Leiden University, Leiden, The Netherlands) was used in order to create the term maps by using the following options: “Create a map based on bibliographic data,” “read data from bibliographic database files,” “type of analysis: co-occurrence,” “unit of analysis: all keywords,” “counting method: full counting,” and “minimum number of occurrence of a keyword: 20.”
