# Supplementary material for: Habenula bibliometrics: Thematic development and research fronts of a resurgent field
Source: Front Integr Neurosci. 2022 Aug 3;16:949162. doi: 10.3389/fnint.2022.949162 (PMC9382245; doi:10.3389/fnint.2022.949162)
Supplement: Supplementary file 2 [file Table_1.docx]

| **Supplementary Table 1** The top 10 countries/regions and institutions contributing to publications in habenula research | | | | | | | | | | |  | |  | |
| --- | --- | --- | --- | --- | --- | --- | --- | --- | --- | --- | --- | --- | --- | --- |
| Rank | Country/  region | Article counts | Total  citations |  | Institutions | Article counts | Total number of citations | Average number of citations | Total number of first authors | Total number of first author citations | Average number of first author citations | Country | |  |
| 1 | USA | 721 | 24636 |  | Icahn Sch Med Mt Sinai | 78 | 668 | 8.56 | 18 | 127 | 7.06 | USA | |  |
| 2 | PEOPLES R CHINA | 214 | 3395 |  | Jilin Univ | 72 | 535 | 7.43 | 34 | 289 | 8.5 | China | |  |
| 3 | JAPAN | 150 | 3218 |  | Heidelberg Univ | 59 | 322 | 5.46 | 17 | 119 | 7 | German | |  |
| 4 | GERMANY | 139 | 2872 |  | Univ Calif San Diego | 56 | 1260 | 22.5 | 18 | 544 | 30.22 | USA | |  |
| 5 | FRANCE | 134 | 2892 |  | Univ Washington | 55 | 565 | 10.27 | 14 | 135 | 9.64 | USA | |  |
| 6 | ENGLAND | 95 | 2633 |  | Stanford Univ | 53 | 1050 | 19.81 | 10 | 233 | 23.3 | USA | |  |
| 7 | SPAIN | 89 | 1340 |  | Xi An Jiao Tong Univ | 46 | 159 | 3.46 | 23 | 75 | 3.26 | China | |  |
| 8 | CANADA | 85 | 1314 |  | Baylor Coll Med | 45 | 288 | 6.4 | 13 | 178 | 13.69 | USA | |  |
| 9 | SOUTH KOREA | 62 | 718 |  | CNRS | 44 | 587 | 13.34 | 12 | 189 | 15.75 | France | |  |
| 10 | ITALY | 61 | 867 |  | Chinese Acad Sci | 37 | 303 | 8.19 | 12 | 140 | 11.67 | China | |  |
