# Supplementary material for: Habenula bibliometrics: Thematic development and research fronts of a resurgent field
Source: Front Integr Neurosci. 2022 Aug 3;16:949162. doi: 10.3389/fnint.2022.949162 (PMC9382245; doi:10.3389/fnint.2022.949162)
Supplement: Supplementary file 3 [file Table_2.docx]

| **Supplementary Table 2** The top 11 most active journals that published articles in habenula research (sorted by count) | | | | |  | | | |
| --- | --- | --- | --- | --- | --- | --- | --- | --- |
| Rank | Journal title | Country | Quartile  in category (2020) | Impact Factor  (2020) | | Article counts | Total number of citations | Average number of citations |
| 1 | JOURNAL OF NEUROSCIENCE | USA | Q1 | 6.167 | | 84 | 1662 | 19.79 |
| 2 | JOURNAL OF COMPARATIVE NEUROLOGY | USA | Q3 | 3.215 | | 77 | 896 | 11.64 |
| 3 | NEUROSCIENCE | USA | Q2 | 3.59 | | 67 | 436 | 6.51 |
| 4 | NEUROPHARMACOLOGY | UK | Q1 | 5.25 | | 52 | 325 | 6.25 |
| 5 | BRAIN RESEARCH | Netherlands | Q3 | 3.252 | | 52 | 267 | 5.13 |
| 6 | NEURON | USA | Q1 | 17.173 | | 43 | 1192 | 27.72 |
| 7 | BEHAVIOURAL BRAIN RESEARCH | Netherlands | Q2 | 3.332 | | 43 | 291 | 6.77 |
| 8 | PLOS ONE | USA | / | 3.24 | | 43 | 183 | 4.26 |
| 9 | SCIENTIFIC REPORTS | UK | / | 4.379 | | 31 | 81 | 2.61 |
| 10 | EUROPEAN JOURNAL OF NEUROSCIENCE | France | Q2 | 3.386 | | 30 | 465 | 15.5 |
| 11 | NEUROPSYCHOPHARMACOLOGY | UK | Q1 | 7.853 | | 30 | 304 | 10.13 |
