# Supplementary material for: Habenula bibliometrics: Thematic development and research fronts of a resurgent field
Source: Front Integr Neurosci. 2022 Aug 3;16:949162. doi: 10.3389/fnint.2022.949162 (PMC9382245; doi:10.3389/fnint.2022.949162)
Supplement: Supplementary file 4 [file Table_3.docx]

**Supplementary Table 3** The top 25 most productive authors contributed to publications in habenula research

| Rank | Author | Article counts | Total number of citations | Average number of citations | First author counts | First author citations counts | Average first author citation counts | Corresponding author | Corresponding author citation counts |
| --- | --- | --- | --- | --- | --- | --- | --- | --- | --- |
| 1 | Zhao, H | 25 | 305 | 12.2 | 1 | 57 | 57 | 19 | 198 |
| 2 | Ye, JH | 23 | 204 | 8.87 | 1 | 0 | 0 | 23 | 204 |
| 3 | Zuo, WH | 22 | 190 | 8.64 | 7 | 80 | 11.43 | 0 | 0 |
| 4 | Zhang, L | 22 | 91 | 4.14 | 2 | 14 | 7 | 2 | 6 |
| 5 | Liu, J | 20 | 75 | 3.75 | 0 | 0 | 0 | 16 | 72 |
| 6 | Mameli, M | 19 | 332 | 17.47 | 0 | 0 | 0 | 14 | 310 |
| 7 | Fu, R | 18 | 133 | 7.39 | 5 | 29 | 5.8 | 0 | 0 |
| 8 | Jhou, TC | 18 | 556 | 30.89 | 4 | 253 | 63.25 | 10 | 291 |
| 9 | Li, J | 17 | 128 | 7.53 | 4 | 35 | 8.75 | 0 | 0 |
| 10 | Halpern, ME | 16 | 276 | 17.25 | 0 | 0 | 0 | 11 | 218 |
| 11 | Salas, R | 16 | 163 | 10.19 | 2 | 122 | 61 | 10 | 34 |
| 12 | Sartorius, A | 16 | 196 | 12.25 | 3 | 75 | 25 | 6 | 149 |
| 13 | Wang, Y | 16 | 64 | 4 | 0 | 0 | 0 | 0 | 0 |
| 14 | Bekker, A | 15 | 140 | 9.33 | 0 | 0 | 0 | 0 | 0 |
| 15 | Hikosaka, O | 15 | 1227 | 81.8 | 1 | 167 | 167 | 3 | 626 |
| 16 | Veh, RW | 15 | 428 | 28.53 | 0 | 0 | 0 | 10 | 315 |
| 17 | Lecca, S | 14 | 323 | 23.07 | 7 | 235 | 33.57 | 1 | 7 |
| 18 | Li, Y | 14 | 66 | 4.71 | 3 | 2 | 0.67 | 1 | 0 |
| 19 | Ogawa, S | 14 | 63 | 4.5 | 3 | 24 | 8 | 3 | 12 |
| 20 | Parhar, IS | 14 | 81 | 5.79 | 0 | 0 | 0 | 9 | 49 |
| 21 | Kenny, PJ | 13 | 145 | 11.15 | 0 | 0 | 0 | 6 | 119 |
| 22 | Guo, Y | 13 | 44 | 3.38 | 2 | 3 | 1.5 | 1 | 0 |
| 23 | Lecourtier, L | 13 | 422 | 32.46 | 6 | 193 | 32.17 | 4 | 40 |
| 24 | Okamoto, H | 13 | 546 | 42 | 1 | 3 | 3 | 12 | 523 |
| 25 | Gamse, JT | 13 | 181 | 13.92 | 2 | 118 | 59 | 6 | 32 |
