# Supplementary figures and images for: Habenula bibliometrics: Thematic development and research fronts of a resurgent field
Source: Front Integr Neurosci. 2022 Aug 3;16:949162. doi: 10.3389/fnint.2022.949162 (PMC9382245; doi:10.3389/fnint.2022.949162)

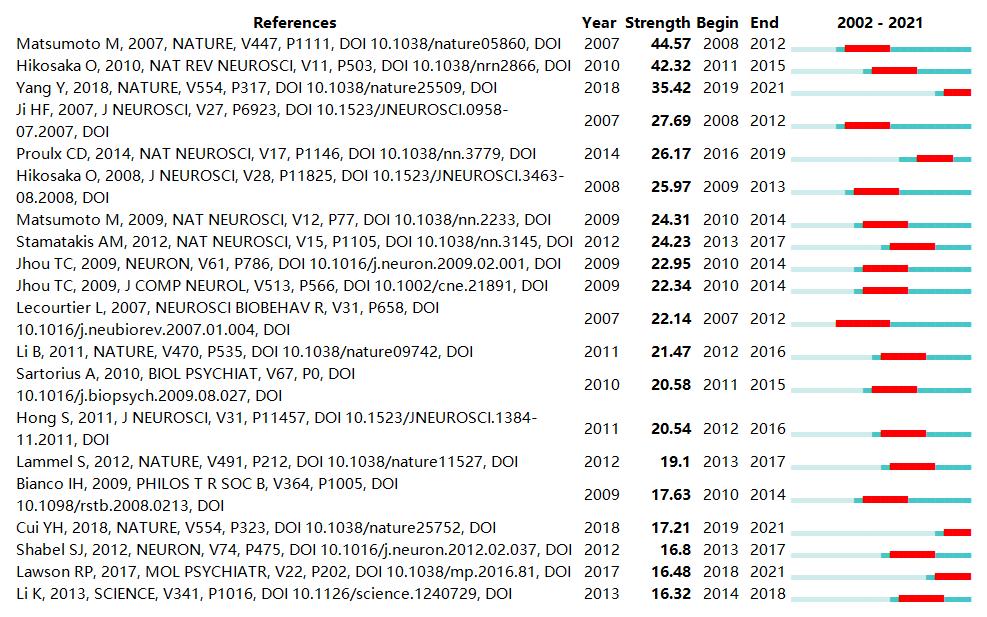

Supplement: Supplementary file 6 [file Image_2.jpeg]
